# Precision nutrition for targeting pathophysiology of cardiometabolic phenotypes

**DOI:** 10.1007/s11154-023-09821-5

**Published:** 2023-07-05

**Authors:** Christopher E. Shannon, Méabh B. Ní Chathail, Sinéad M. Mullin, Andrew Meehan, Fiona C. McGillicuddy, Helen M. Roche

**Affiliations:** 1 Nutrigenomics Research Group, UCD Conway Institute, and Institute of Food and Health, School of Public Health, Physiotherapy and Sports Science, University College Dublin, Dublin, Republic of Ireland; 2 Institute for Global Food Security, Queen’s University Belfast, Belfast, Northern Ireland; 3 School of Medicine, University College Dublin, Dublin, Republic of Ireland; 4 Division of Diabetes, Department of Medicine, UT Health San Antonio, San Antonio, TX USA

**Keywords:** Precision nutrition, Obesity, NAFLD, Insulin resistance, Inflammation

## Abstract

Obesity is a heterogenous disease accompanied by a broad spectrum of cardiometabolic risk profiles. Traditional paradigms for dietary weight management do not address biological heterogeneity between individuals and have catastrophically failed to combat the global pandemic of obesity-related diseases. Nutritional strategies that extend beyond basic weight management to instead target patient-specific pathophysiology are warranted. In this narrative review, we provide an overview of the tissue-level pathophysiological processes that drive patient heterogeneity to shape distinct cardiometabolic phenotypes in obesity. Specifically, we discuss how divergent physiology and postprandial phenotypes can reveal key metabolic defects within adipose, liver, or skeletal muscle, as well as the integrative involvement of the gut microbiome and the innate immune system. Finally, we highlight potential precision nutritional approaches to target these pathways and discuss recent translational evidence concerning the efficacy of such tailored dietary interventions for different obesity phenotypes, to optimise cardiometabolic benefits.

## Background

It is projected that by the year 2030 one billion individuals will be living with overweight or obesity worldwide (World Obesity Atlas, 2022). This, paralleled by rising rates of obesity-related diseases such as type 2 diabetes, non-alcoholic fatty liver and cardiovascular disease, presents a monumental public health concern. Accordingly, effective therapeutic strategies for the prevention and treatment of obesity have tremendous socioeconomic value. Whilst bariatric surgeries and recent generations of incretin mimetics offer significant advances in anti-obesity therapies [1, 2], dietary modification remains an integral component of effective obesity treatment paradigms.

Considerable inter-individual differences exist with respect to the health impacts of a surplus energy balance, presenting a significant challenge in predicting which (if any) obesity-related complications may develop in response to chronic weight gain. Moreover, the same phenotypic heterogeneity is paralleled by extensive variability in patient responses to interventions designed to improve metabolic parameters including insulin resistance and dyslipidaemia [3]. Traditionally, anti-obesity therapies have targeted excess body weight as the underlying pathophysiological feature of the disease. Notably, many obesity-related complications require >10% weight loss to observe meaningful clinical benefit [4, 5] and, although weight losses of 20-30% may now be obtainable with surgical [6] or pharmacological [7] treatments, intensive lifestyle interventions typically only achieve weight losses of 5-10% [8, 9]. Furthermore, the modest benefits of weight loss following dietary intervention [5] are largely unsustainable, since most patients regain the majority of this weight over a five year period [10].

Given the vast pathophysiological diversity of obesity and its associated conditions, it is perhaps unsurprising that generic “one-size fits all” dietary approaches exclusively focused on optimising weight control have thus far failed to curb escalating rates of obesity-related disease. Accordingly, nutritional strategies that go beyond basic weight management to instead target phenotypic pathophysiology are warranted. In this narrative review we discuss current understanding of the biological pathways underpinning patient heterogeneity in obesity and consider recent evidence that targeted nutritional modulation of these pathways can improve the cardiometabolic health of patients .

### Moving beyond GWAS

Despite extensive progress over the past 15-20 years of gene-wide association studies (GWAS) identifying countless monogenic or polygenic modifiers of obesity risk [11, 12], the biological pathways linking genetic variability to body weight regulation remain largely unresolved. Moreover, there is a growing appreciation that the diverse metabolic phenotypes of obesity cannot be ascribed to genetic variation alone, suggesting that environmental factors are key additional determinants of metabolic diversity and subsequent disease risk. Precision medicine paradigms strive to improve patient outcomes by optimising treatment strategies based upon genetic, epigenetic, metagenetic, metabolic, or psycho-behavioural characteristics. Conceptual and technological advances in the stratification of patients with obesity with respect to these characteristics are discussed elsewhere in this Issue of Reviews in Endocrine and Metabolic Disorders. Translating this phenotypic patient information towards personalised nutrition recommendations requires both an understanding of the underlying pathophysiology driving different obesity phenotypes, as well as robust evidence for nutritional strategies that correct these dysfunctional pathways.

One of the core pathophysiological features of obesity is metabolic dysfunction. However, the relationship between weight gain and metabolic decline is nonlinear, such that for any given body mass index (BMI) or body fat percentage there exists a continuum of cardiometabolic risk profiles [13]. For example, this spectrum includes a cohort of individuals who, despite being obese, present as metabolically healthy (MHO) and, compared to metabolically unhealthy patients with obesity (MUO), appear relatively protected (although perhaps only transiently) from type 2 diabetes, cardiovascular disease and chronic kidney disease [14]. Nevertheless, obesity *per se* still confers an increased risk of cardiometabolic diseases in individuals who are metabolically healthy, and up to 50% of individuals with MHO were found to progress to MUO over a four-year period [14, 15].

**Fig. 1:**
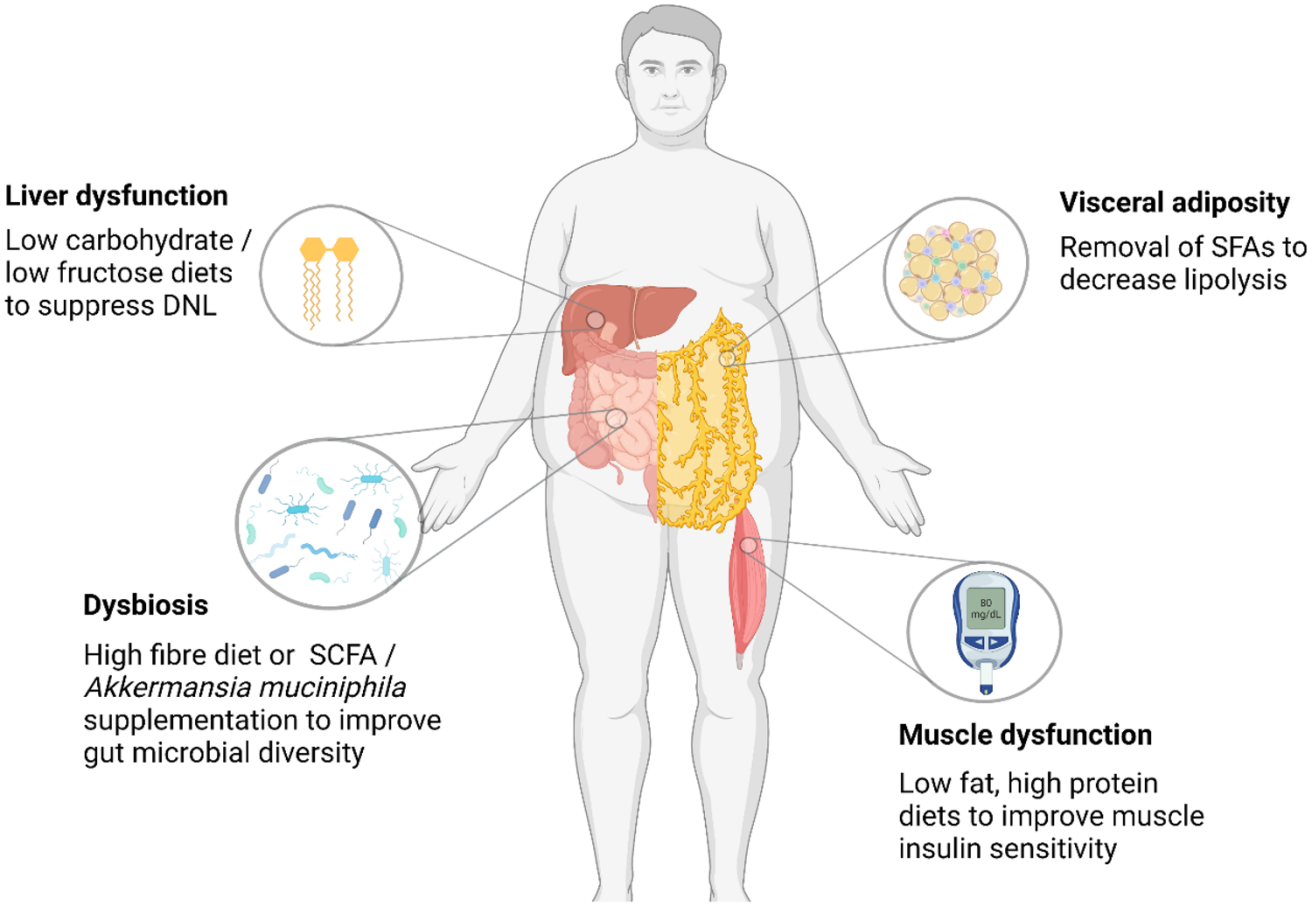
Overview of the potential nutritional strategies to target tissue-specific pathophysiology in different obesity phenotypes

The extent and precise nature (e.g. tissue-specificity) of metabolic dysfunction has important implications for the natural history of obesity phenotypes and, by extension, for the development of targeted treatment approaches. Indeed, metabolic reprogramming has recently emerged as an important determinant of an individual’s response to dietary intervention [16]. In the following sections we will discuss tissue-specific metabolic disturbances implicated in the pathogenesis of the major obesity phenotypes (Fig. 1), highlighting potentially targetable pathways for the design of evidence-based precision nutrition strategies.

## Adipose tissue phenotypes to guide precision nutrition

### Adipose tissue dysfunction

Obesity exemplifies the plasticity of adipose tissue as the primary site in the body for energy storage. In extreme cases adiposity can exceed 50% of total body mass [17], equating to an energy reservoir of nearly two gigajoules. Despite this remarkable capacity for expansion, chronically increased nutrient storage ultimately compromises adipocyte function and disrupts the integration of whole-body metabolism. Excessive adiposity drives the increased delivery of non-esterified fatty acids (NEFA) to non-adipose tissues, promoting ectopic lipid accumulation and peripheral insulin resistance [18]. Enlarged adipocytes also become progressively pro-inflammatory [19] which, perpetuated by pathogenic interactions with stromal vascular cells [20], contributes to the exacerbation of whole-body insulin resistance and obesity-related disease progression [21].

The accumulation of adipose tissue in visceral depots, as opposed to subcutaneous depots, is especially deleterious to metabolic health, since untapped lipolysis from insulin-resistant perivisceral adipocytes exposes metabolic tissues to high burdens of lipotoxic compounds such as saturated fatty acids and ceramides [22, 23]. Accordingly, sexual dimorphism in adipose distribution (females tend to accumulate less visceral adipose than BMI-matched males) likely contributes to the increased incidence of obesity-related disease in males versus females [24]. Interestingly, up to one third of the loci associated with adipose tissue distribution display a strong sex-dependence [25], providing genetic insight into the increased propensity for visceral adipose accumulation (and cardiometabolic disease risk) in males versus females. As will be discussed further below, the role of biological sex in shaping adipose tissue phenotypes has important implications for understanding the sex-dependent responses to dietary intervention and the subsequent design of precision nutrition approaches.

In concert with sex-related differences, variable constraints on the expandability of subcutaneous adipose tissue may also explain why certain individuals are phenotypically prone to increased visceral adipose accumulation [26]. Notably, many of the genetic loci differentiating between MHO and MUO are found near genes involved in the regulation of adipogenesis and fat mass distribution [27]. Recent support for this concept comes from a study separating individuals of an “obesogenic genotype” into two extensively phenotyped cohorts, based upon their genetic concordance (or discordance) for type 2 diabetes [28]. The phenotypic traits distinguishing these two cohorts included traditional indices of cardiometabolic risk (waist-to-hip ratio, dyslipidemia, hypertension, and liver enzymes), as well as other previously identified serum biomarkers of metabolic dysfunction (branched chain amino acids, phosphatidylcholines). However, of the discordant genetic variants identified, the master adipogenesis regulator PPARγ emerged as the key modifier of the relationship between obesity and diabetes risk, underlining the link between adipose expandability and metabolic health. Therapeutic targeting of this axis will be discussed in relation to “*Precision nutrition to compliment adjunct pharmacotherapies*”.

### Impact of adipose dysfunction on postprandial responses

Since humans spend the majority of waking hours in the fed state, postprandial physiology (i.e. the hormonal, metabolic and inflammatory responses to food) acts as a powerful driver of obesity-related disease progression. Indeed, postprandial responses to nutrient challenges are strong predictors of future cardiometabolic disease risk [29–31] and capture a greater fraction of patient phenotypic heterogeneity than relying on fasting indices alone [32]. In healthy adipocytes, insulin-mediated glucose uptake, plasma triglyceride hydrolysis and fatty acid esterification all promote triglyceride synthesis to buffer postprandial increases in systemic nutrient availability [33, 34]. Sophisticated *in vivo* measurements of postprandial lipid fluxes demonstrate that, whilst the capacity for disposing dietary fatty acids within adipose tissue is increased in individuals with obesity, it remains insufficient to fully offset the impaired suppression of postprandial lipolysis from insulin-resistant adipocytes [35]. The resulting “lipid overspill” during postprandial periods accelerates fat deposition in visceral adipose tissue [26] and other non-adipose organs [36]. The association between lipid overspill and visceral adipose accretion is further highlighted by observations that visceral adiposity predicts the acute postprandial triglyceride response to a high fat meal [37–39] and is strongly related to changes in ectopic fat accumulation following chronic (8 weeks) overfeeding [40]. As such, postprandial insulin responses and/or hyperlipidemia following a standardised nutrient challenge (high-fat or mixed meal) may offer a useful metric by which to identify individuals with dysfunctional adipose tissue and at a high risk of obesity-related disease progression.

### Nutritional modulation of adipose dysfunction

Overfeeding saturated fat drives adipose insulin resistance and increased lipolysis [41], and promotes further preferential expansion of visceral adipose depots [42], leading to an exacerbated dietary cycle of lipotoxicity in metabolic tissues. Additionally, hypercaloric diets enriched in fructose (but not glucose) have also been shown to drive increases in visceral adiposity, as an apparent consequence of fructose-induced postprandial hypertriglyceridemia [43]. These data provide a link between fructose-mediated hepatic *de novo* lipogenesis and visceral fat storage, demonstrating the bidirectional relationship between metabolic pathophysiology within the adipose tissue and liver. Interestingly, visceral (intrabdominal) fat accretion following fructose overfeeding was greater in males than females, whereas decreases in insulin sensitivity were more marked in females [43], thus reaffirming the importance of biological sex in mediating the metabolic effects of certain dietary triggers.

Based upon findings from overfeeding studies [40–43] and the discussion above it might be speculated that individuals with dysfunctional adipose tissue, as identified from phenotypic disturbances in postprandial lipid metabolism, could be especially sensitive to the removal of dietary saturated fats and/or fructose. Indeed, large-scale studies in which patients were retrospectively stratified by their response to dietary intervention indicate that baseline metabolic phenotypes (e.g. insulin sensitivity) may influence the effects of dietary fat modification [44]. In the DiOGenes study, sex-specific differences in adipose tissue distribution were associated with the superior metabolic benefits gained by males versus females following a low-calorie diet [45]. Recent preliminary data further suggest that both sex and age requires careful consideration for the development of precision nutrition strategies [46]. In response to four weeks of caloric restriction, younger (<45 years) males lost more body fat than age-matched females, whereas changes in fat mass in older (>45 years) individuals were equivalent between sexes. In contrast, older males lost more lean mass than age-matched females which, as will be discussed, has important implications for the management of sarcopenic obesity in this population.

A final potentially targetable pathway in adipose tissue is the induction of thermogenesis as a means of increasing energy expenditure [47]. Probably the most convincing evidence that adipose tissue thermogenesis may appreciably influence whole body energy expenditure in humans comes from studies of acute burn trauma. In such patients, increases in uncoupling protein one (UCP1)-dependent energy turnover can be attributed to both the sustained activation of brown fat and the so-called browning of white adipose depots [48]. Whereas impaired brown fat metabolism has been implicated in the pathogenesis of obesity-related disease [49–51], translational evidence linking dietary modification with adipose tissue energy expenditure in humans is lacking. Interestingly however, it was recently demonstrated that overfeeding fructose (but not glucose) in healthy humans during a short-term (two weeks) high carbohydrate diet impaired glucose uptake into brown adipose tissue, prior to other changes in cardiometabolic parameters [52]. The potential for precision nutrition targeting of adipose thermogenesis will undoubtedly be the subject of intense scrutiny and future research investigation.

## Insulin resistance phenotypes to guide precision nutrition

### Peripheral insulin resistance predicts response to dietary intervention

As discussed above, inadequate lipid buffering by dysfunctional subcutaneous adipose tissue promotes fat storage in both visceral adipose depots and non-adipose organs. Ectopic lipid accumulation is a key driver of insulin resistance, one of the earliest detectable hallmarks of MUO [53]. The product of fasting glucose x fasting insulin (HOMA-IR) is routinely used to stratify individuals with obesity by their degree of insulin resistance and has been shown to be predictive of individual responses to dietary intervention [54]. In the LIPGENE study, involving a group of 472 individuals with metabolic syndrome, increased HOMA-IR was associated with a greater sensitivity to the metabolic effects of reducing dietary saturated fats [44]. In a similar cohort of 639 subjects, the POUNDS LOST Trial reported that HOMA-IR retrospectively predicted whether individuals were more likely to respond to a low fat/high protein, a high fiber, or a high fat/high protein diet [55]. More recently, when comparing the efficacy of high-nutrient quality (low fructose, enriched in fiber, soy protein and unsaturated fatty acids) versus low-nutrient quality energy-restriction, the Wageningen Belly Fat Team found that differences between the metabolic benefits of each diet were more pronounced for individuals with lower HOMA-IR [56].

### Postprandial glucose metabolism reveals distinct pathophysiology

The cumulative evidence described above provided initial justification for investigating whether metabolic phenotyping could be used to prospectively guide optimized nutritional strategies in randomized controlled trials. However, consistent with the complex and multi-faceted pathogenesis of insulin resistance, it must be acknowledged that associations between fasting HOMA-IR and dietary efficacy are strongly influenced by covariates such as biological sex, weight and age. Patterns of postprandial glucose excursion can provide more detailed information about insulin resistance pathophysiology, potentially allowing for the design of more targeted nutritional approaches. For example, since skeletal muscle represents the primary site of insulin-mediated glucose uptake, accounting for 60-70% of postprandial glucose disposal [57], insulin resistance in skeletal muscle manifests as reductions in postprandial glucose clearance. In contrast, hepatic insulin resistance primarily manifests as an increase in fasting glucose levels (and in the ascending limb of the postprandial glucose excursion curve), due to the inability of insulin to switch off liver glucose output from both glycogenolysis and gluconeogenesis [58].

Using the conceptual framework outline above, individuals with obesity have been phenotypically characterized as insulin resistant at either the muscle (impaired glucose tolerance, IGT) or liver (impaired fasting glucose, IFG), or both (IGT/IFG) based on their glycemic response to an oral glucose tolerance test [59]. In a European cohort of 634 patients with overweight, a recent study reported incidences of 14, 16 and 17% for isolated IGT, isolated IFG or IGT/IFG, respectively [60], whereas earlier studies have estimated the prevalence of IGT or IFG to be as high as 20-25% in some countries [61]. Women are more likely to stratify as IGT, whereas IFG appears to be more common in men [60], indicating a sexually dimorphic manifestation of tissue-specific insulin resistance. Interestingly, data from the CORDIOPREV trial suggests that liver specific insulin resistance may drive increases in postprandial lipids, with IFG patients more likely to display hypertriglyceridemia after a high fat meal challenge [62]. These data further highlight how metabolic crosstalk between the liver and adipose tissue modulates postprandial lipid levels to drive obesity phenotypes. Overall, IGT and IFG display diverging comorbidity risk profiles [63] and are associated with distinct metabolic abnormalities [60, 64, 65].

### Precision nutrition strategies to target insulin resistance phenotypes

The phenotypic differences between IGT and IFG have important implications for the implementation of targeted nutrition interventions in these populations. Leveraging this concept, Trouwborst and colleagues recently demonstrated that individuals with muscle-specific insulin resistance (IGT glycemic response) showed greater metabolic improvements on a low fat, high fiber diet compared to an isocaloric diet enriched in monounsaturated fat [66]. Conversely, the same low-fat, high fiber diet was less beneficial for individuals with liver-specific insulin resistance (IFG glycemic profile), who were instead more responsive to the monounsaturated fat diet. This study represents a significant step towards the practical application of precision nutrition paradigms and yet, interestingly, the findings opposed the authors initial hypotheses based upon their previous observations [67]. As such, these results highlight an important translational gap in our understanding of the relationship between insulin resistance phenotypes and dietary responses.

#### Nutritional modulation of hepatic insulin resistance

One of the major pathological mechanisms underpinning hepatic insulin resistance is the accumulation of intrahepatic triglycerides, precipitating the onset of non-alcoholic fatty liver disease (NAFLD) and, in a subset of individuals with obesity, progression to the inflammatory, fibrotic condition of steatohepatitis (see section on NAFLD). Hepatic lipid accumulation is driven both by increases in lipid overspill from dysfunctional adipose tissue (previously discussed) and by the sustained activation of hepatic *de novo* lipogenesis (DNL). High carbohydrate diets, especially those enriched in fructose and / or sucrose [43, 68], have been shown to promote hepatic DNL, whereas polyunsaturated fatty acid supplementation may suppress DNL [69–71]. Recent data also suggests that constituently activated DNL in a subset of patients with obesity is associated with hepatic mitochondrial dysfunction and impaired ketogenic disposal of lipid oxidation-derived acetyl-CoA [72]. Interestingly, a short term (one week) ketogenic diet has been shown to redirect fatty acid partitioning towards ketone synthesis and alleviate mitochondrial oxidative fluxes [73], further supporting the use of carbohydrate restriction to reverse metabolic dysfunction in the liver. Finally, an impressive reduction in intrahepatic triglycerides was recently reported in patients with obesity administered a polyphenol-enriched Mediterranean diet versus a Mediterranean diet alone [74], offering another potential nutritional strategy to combat hepatic insulin resistance in affected individuals. Further RCTs, like the study of Trouwborst et al., comparing the efficacy of these approaches between IFG and IGT phenotypes are required to develop actionable precision nutrition programmes.

#### Nutritional modulation of skeletal muscle insulin resistance

Much like the accumulation of intrahepatic triglycerides in the liver, the accretion of toxic lipid metabolites such as diacylglycerides [75] and ceramides [76] is one of the best-studied mechanisms implicated in the development of skeletal muscle insulin resistance. In addition to surplus muscle lipid delivery from adipose, deficiencies in the intrinsic capacity for fatty acid oxidation (i.e. mitochondrial dysfunction) have also been reported in the skeletal muscle from individuals with obesity [77]. Although the direct causative role for mitochondrial dysfunction in the development of skeletal muscle insulin resistance remains contentious, strong rational exists for the nutritional targeting of muscle lipid oxidation in obesity, especially for individuals with IGT or muscle-specific insulin resistance. Numerous dietary strategies (e.g. intermittent fasting) and bioactive compounds (e.g. resveratrol, coenzyme Q10) have been proposed to improve mitochondrial function and lipid oxidation in skeletal muscle. However, convincing support from translational human trials is currently sparse [78]. As mentioned, a low fat, high protein and high fiber diet was found to be more beneficial than a high monounsaturated fat diet in individuals with muscle insulin resistance [66], although the mechanisms responsible for this remain unclear.

One promising nutritional approach to enhance muscle lipid oxidation is through the manipulation of free carnitine availability in skeletal muscle. Carnitine is required for the mitochondrial entry of long-chain fatty acids for subsequent β-oxidation and daily L-carnitine supplementation has been shown to enhance intramyocellular lipid oxidation by ~20% [79]. Moreover, a carnitine-mediated increase in whole-body fat oxidation appears to be protective against body fat gain during prolonged periods (six months) of carbohydrate overfeeding [80]. To date, dietary strategies to increase muscle carnitine content have required the daily consumption of L-carnitine with insulinogenic (and moderately calorific) beverages for 12-24 weeks, making it impractical for use in obese, insulin resistant populations. However, very recent data suggest that dietary caffeine may offer an effective alternative to insulinotropic macronutrients for stimulating muscle carnitine retention [81], paving the way for future investigations into the benefits of carnitine loading as a means of targeting muscle lipid oxidation in individuals with obesity with muscle specific insulin resistance.

In addition to precipitating IGT phenotypes, skeletal muscle insulin resistance can be associated with an impaired anabolic response to amino acid feeding, leading to a blunting of muscle protein synthesis [82] and which, in extreme cases, can present as an overall loss of lean mass. In the context of excess adiposity this loss of muscle, termed sarcopenic obesity, exacerbates the progression of metabolic decline and further compounds the disease risks associated with increased body fat alone [83]. Nutritional strategies targeting sarcopenia as an isolated pathophysiology typically include a high proportion of dietary protein and essential amino acids (particularly leucine), which act as potent anabolic stimuli to promote a positive muscle protein balance [84]. Recent studies also support the avoidance of very low-calorie diets in sarcopenic obesity, which are often prescribed to promote weight loss in individuals with obesity but may exacerbate the loss of lean mass even in the presence of increased protein intake [85]. As described above, older males may be at heightened risk of muscle loss following caloric restriction [46] and so alternate strategies to tackle obesity are likely to be more appropriate for this population.

Several studies have attempted to combine protein feeding with other micronutrients in sarcopenic risk groups, to potentiate the anabolic effects of amino acids and overcome anabolic resistance. For example, omega-3 polyunsaturated fatty acids (PUFA) have been reported to protect against declines in muscle mass and strength in older adults [86]. The pleiotropic mechanisms through which n-3 PUFA may counter sarcopenia are believed to include anti-inflammatory or pro-resolving pathways [87], as well as the mitigation of lipid-induced insulin resistance [88]. However, evidence for the beneficial effects of n-3 PUFA in human skeletal muscle is conflicting. Indeed, we recently observed no impact of n-3 PUFA plus leucine supplementation on muscle mass, strength, or muscle protein synthesis in a randomized placebo-controlled trail of 107 older adults at risk of sarcopenia [89]. A major challenge for precision nutrition is in identifying which individuals with obesitymay be at greatest risk of sarcopenia, and robust biomarkers to stratify patients with obesity most likely to benefit from dietary interventions targeting muscle mass preservation are currently lacking.

## Precision nutrition management of non-alcoholic fatty liver disease

**Fig. 2:**
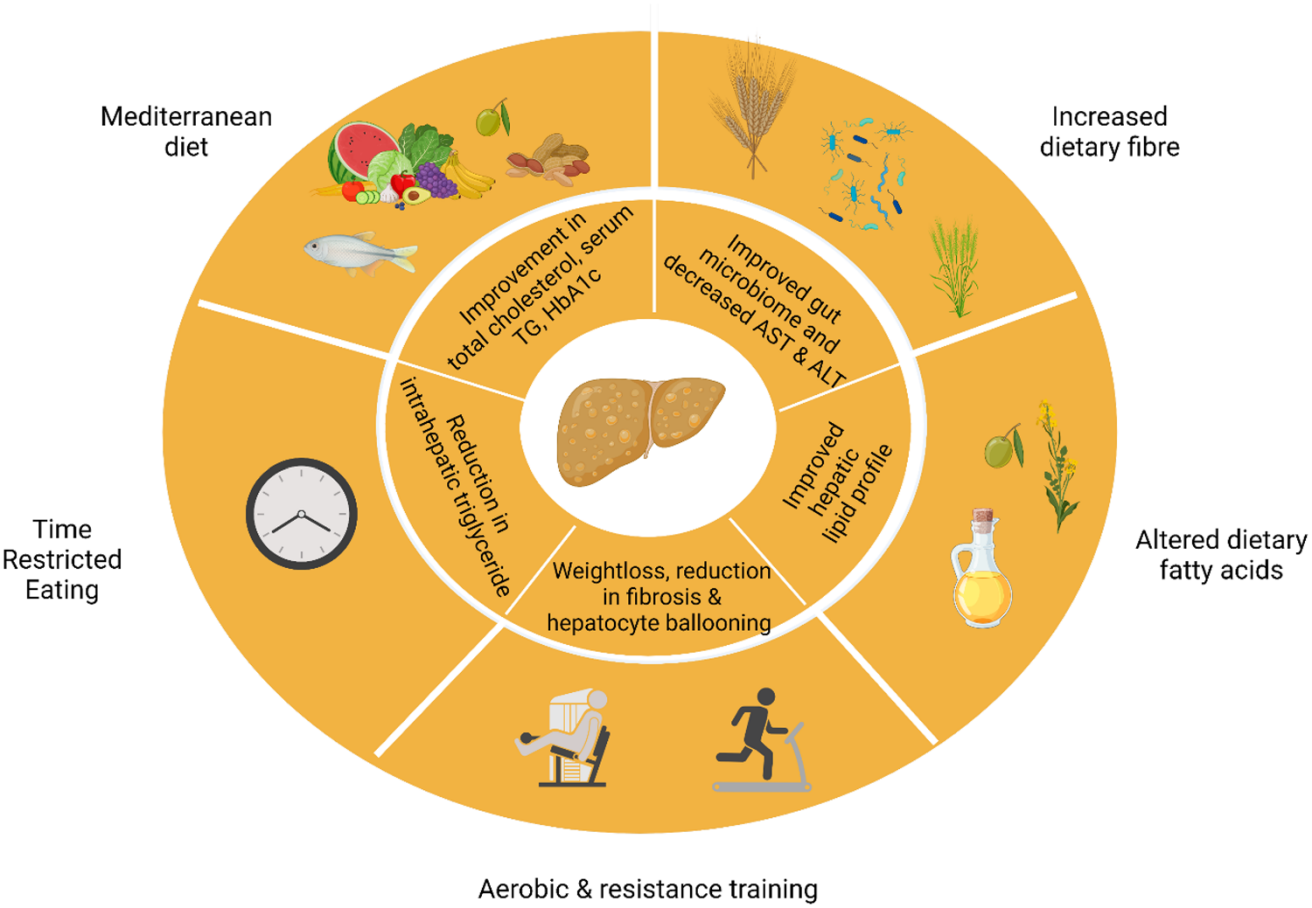
Precision nutrition management of non-alcoholic fatty liver disease

One of the primary phenotypic complications of obesity is the development of non-alcoholic fatty liver disease (NAFLD) which is characterized by an accumulation of triglycerides in hepatocytes (steatosis) [90]. NAFLD is now one of the most common chronic liver disorders worldwide and affects an estimated 25% of the global population [91]. Obesity increases the risk of NAFLD development 3.5-fold relative to normal weight controls [92]. NAFLD in turn is a spectrum of disease which ranges from simple steatosis to non-alcoholic steatohepatitis (NASH) to irreversible liver fibrosis/cirrhosis often culminating in liver failure [90]. NASH, which is characterized by advanced inflammation and hepatocyte ballooning, is a particularly critical disease stage to identify in a timely manner as at this point damage to the liver is reversible [93]. A recent meta-analysis reported that the prevalence of NAFLD in people living with obesity was 75% with NASH prevalence estimated to be 34% [94]. The prevalence of liver complications exceeds the prevalence of other complications of obesity, including type 2 diabetes, making it a particularly critical demographic to better identity and manage. Indeed, NASH is now the most common indication for liver transplantation in women and is predicted to become the most common indication across all patients in the coming years [95]. Furthermore, prevalence of cardiovascular death is also higher in patients with NASH (15.5%) compared to the general population (7.5%) [96]. Despite the global rise in obesity and associated liver disease, there are currently no approved pharmacological interventions to manage NAFLD and/or NASH [97]. There have been several hurdles impeding effective management of patients with NAFLD including the lack of non-invasive biomarkers to detect NASH at a population level, poor understanding of disease pathophysiology, remarkable difficulties in translating findings from mice to man [98] and considerable heterogeneity in response to any given drug [97]. There is therefore a particularly critical role for Precision nutrition approaches to manage NAFLD/NASH due to the current void of pharmacotherapies for these high-risk patients. Dietary strategies that have demonstrated potential for targeting NAFLD pathophysiology, illustrated in Fig. 2, are discussed below.

### Importance of weight-loss for NAFLD management

Caloric restriction, and associated weight-loss, in patients with NAFLD is a particularly effective approach to attenuate the burden of NAFLD. Weight-loss reductions of >10% can mediate NASH resolution and fibrosis improvement by at least one stage [99, 100]. The degree of weight reduction is one of the strongest determinants of reductions in liver fat and /or liver alanine aminotransferase (ALT) concentration in patients with NAFLD [100]**.** Weight-loss via bariatric surgery is similarly very effective at treating NASH with resolution of the disease evident in 85% of surgical patients with Class III obesity after 12 months (*n*=109) [101]. Patients whose NASH persisted within this cohort lost significantly less weight compared to patients whose NASH resolved. There is also considerable evidence that new state-of-the-art weight-loss drugs including GLP-1 agonists exert both weight-loss dependent and independent effects on liver metabolism to improve NASH in pre-clinical studies as reviewed [102]. Small phase 2 clinical trials with Liraglutide demonstrated resolution of NASH at 48 weeks in 9 of 23 patients (39%), compared with 9% who received placebo [103]. Larger scale investigations for Semaglutide (another GLP-1 agonist) in NASH have been less impressive [104], despite greater weight loss efficacy compared with Liraglutide [105]. Nevertheless, evidence from nutritional, surgical [106] and pharmacological studies highlight the important role for weight-loss, and associated changes in hepatic metabolic pathways, as a target to attenuate NASH. Importantly however, these costly interventions are not accessible for the majority of patients and weight-loss through diet and lifestyle is extremely difficult to attain and even more difficult to sustain [10]. Precision nutrition approaches targeting liver metabolism through diet and lifestyle, independent of weight-loss, may therefore offer additional / superior potential in managing patients with NAFLD/NASH.

### Precision nutrition targeting beyond weight-loss for NAFLD

Given the dominant role of excess accumulation of fatty acids in NAFLD, nutritional approaches aimed at increasing oxidation of hepatic fatty acids is a promising approach to manage liver disease progression. Strategies to achieve this, independent of enforcing a caloric deficit, include dietary macronutrient manipulation, time-restricted feeding (TRF) and exercise which will be reviewed within this section. General nutritional approaches to manage patients with NAFLD have been discussed extensively elsewhere [100, 107, 108].

#### Dietary Macronutrient manipulation for NAFLD management

##### High fibre intervention

NAFLD is associated with significant dysfunction in the constitutional composition of the intestinal microbiome and its associated metabolites. High-fibre intake therefore has been studied as a potential mechanism to improve the microbiome and in turn NAFLD. High fibre intervention via bread replacement with high-fibre buns for two months (*n*=27) was associated with improvements in NAFLD coincident with changes in circulating serum microbiome metabolites [109]. Similarly whole-grain intervention (12 weeks) in patients with NAFLD (*n*=112 randomly assigned into two groups), was associated with a significant reduction in NAFLD grade and ALT, AST and γ-glutamyltransferase levels independent of any changes in anthropometric measurements [110]. These findings indicate that dietary enrichment with fibre is an achievable approach to managing patients with NAFLD.

##### Dietary fatty acids

Epidemiological studies have demonstrated that low dietary intake of polyunsaturated fatty acids (PUFA) and fibre and high dietary intake of saturated fatty acid (SFA) and cholesterol is evident in patients with NASH relative to age-, BMI, and sex-matched controls [111]. A meta-analysis of 4 studies (*n*=263 children), demonstrated that PUFA supplementation was associated with improved hepatic steatosis on ultrasound, with reduction in AST levels at 6 months and ALT levels by 12 months [112]. Another intervention assessing the combined effect of n-3 fatty acids (EPA and DHA) and phytosterol esters (PS) on NAFLD (*n*=96 patients) demonstrated that combination of nutritional supplements exerted more profound effects of the liver:spleen ratio (-36%) relative to either intervention alone (EPA+DHA alone; -18% and PS alone; -11%) [113]. These studies provide promising evidence for a beneficial role of PUFA and phytosterols in managing NAFLD.

The Mediterranean diet has also proven to be beneficial in the management of NAFLD. Intervention with a low glycaemic index Mediterranean diet (*n*=96 total) was negatively associated with NAFLD score at 6 months despite no difference in weight-loss relative to the control arm of the intervention [114]. Properzi et al. [115] have also demonstrated that *ad libitum* isocaloric intervention with both Mediterranean diet (MD) and low-fat (LF) diet for 12 weeks reduced hepatosteatosis and liver enzymes with no difference in liver fat content evident between groups (*n*=48 total). Minimal weight-loss was evident with these interventions and did not differ across groups. The MD, but not LF diet, was also associated with significant improvements in total cholesterol, serum TG and HbA1c. This study found much greater adherence to the MD compared to the LF (88% vs. 64%) which will have important implications for wider acceptability of such a dietary approach at a population level.

#### Time-restricted feeding (TRF) efficacy in NAFLD

While TRF is gaining popularity in facilitating weight-loss by aiding caloric restriction, it also offers a particularly important approach to manipulate metabolism within the liver of patients with NAFLD, ensuring sufficient fasting time to enter substantial fatty acid oxidation coupled with reductions in plasma insulin levels. In a recent study, participants with NAFLD were assigned to TRF (eating only between 8am and 4pm, *n*=45) or daily caloric restriction (DCR, *n*=43) with both groups advised to restrict calories for 12 months. Both intervention arms were associated with significant reductions in intrahepatic triglyceride (IHTG) content with no additional benefit of TRF beyond caloric restriction alone [116].

The findings above highlight a dominant effect of caloric restriction, and no advantage to TRF in the setting of significant caloric deficit, but this does not infer lack of efficacy of TRF in the setting of *ad libitum* feeding. Indeed, fasting during Ramadan was associated with significant reductions in BMI, NAFLD fibrosis scores, FIB4 scores and CRP levels as well as improvements in HOMA-IR in patients with NASH (*n*=74 fasting, *n*=81 no fasting) indicating positive effects of TRF in the absence of enforced caloric restriction [117]. Another study (*n*=271) compared the impact of alternative-day fasting (ADF), TRF (8h feeding; 16h fasting) or control (80% of energy needs without any restriction on usual lifestyle patterns) on the body weight and lipid profiles of patients with NAFLD. Significant weight-loss was observed with both ADF (-11 ± 1.2%) and TRF (-9.6 ± 1.3%) at 12 weeks compared to the control group. ADF had more profound effects on total cholesterol levels compared to both control and TRF groups. ADF therefore appeared to be superior for management of NAFLD-associated dyslipidemia in a short time-frame [118]. The impact on liver fat content was not reported. These findings highlight that TRF has beneficial effects on liver metabolism but whether weight-loss independent effects exist in humans is unknown. In preclinical studies, TRF in the absence of significant changes in caloric intake protected against obesity, hyperinsulinemia, hepatic steatosis and inflammation with changes in catabolic and anabolic pathways evident within the liver metabolome and improved nutrient utilization and energy expenditure evident [119]. Future studies of TRF in the setting of weight neutrality will be very important to elucidate novel nutrient-mediated therapeutic mechanisms in humans.

#### Exercise as an adjunct to dietary intervention in NAFLD

Exercise remains a critical cornerstone in the management of patients with NAFLD. A 12-week aerobic exercise intervention in patients with NAFLD (*n*=16 in exercise group, *n*=8 in control group) demonstrated a significant reduction in fibrosis and hepatocyte ballooning by one stage in 58% and 67% of patients respectively, with no changes in steatosis, lobular inflammation or NAFLD activity score. Reductions in BMI, waist circumference and fat mass were also observed but no patient achieved >7% weight-loss demonstrating weight-independent benefits of exercise in patients with fatty liver disease [120]. A systematic review covering 23 studies found consistent reductions in liver fat and/or liver enzyme concentrations, with the strongest correlation being with weight reduction [99]. A further systematic review sought to compare the effects of aerobic versus resistance exercise in NAFLD (24 aerobic studies and 7 resistance studies selected). Both aerobic and resistance exercise were associated with improved hepatic steatosis however %VO_2_ max and energy consumption were significantly lower in the resistance group than the aerobic group. This study concluded that resistance exercise offers an alternative approach for patients with NAFLD who cannot tolerate aerobic exercise [121]. Babu et al. [122] have more recently conducted a systemic review and meta-analysis investigating the effects of exercise without dietary changes on NAFLD-related clinical parameters (10 studies, with *n*=316 individuals). This study found that exercise without significant weight-loss could significantly reduce intrahepatic lipid content (IHL), ALT, AST, LDL-C and TG levels with no impact on measures of glucose metabolism. Aerobic exercise alone reduced IHL, ALT and AST while resistance alone reduced total cholesterol and TG with combination of both having a significant impact on IHL.

### Looking to the future in NAFLD

Overall these promising findings highlight important precision nutrition approaches that could be implemented in patients with NAFLD that do not solely rely on successful weight-loss for positive outcomes on disease status. There are still some major gaps in knowledge in the field in relation to effective interventions across the liver disease spectrum. Most studies to date have been performed in patients with simple steatosis - more targeted interventions in higher-risk patients with NASH are extremely limited. This is largely due to the lack of biomarkers to identify suitable trial participants for NASH intervention studies. Precision nutrition interventions targeting more clinically meaningful NAFLD subgroups (eg. with/without inflammation and/or fibrosis) will be critical to evaluate in the future to optimize patient management. Indeed, the timely emergence of new blood-based biomarkers for patients across the NAFLD spectrum will be essential to guide precision nutrition interventions [123].

## Microbiome enterotypes to guide precision nutrition

### Microbiome dysbiosis is associated with cardiometabolic disease

We have so far considered how heterogeneous tissue dysfunction in adipose, liver and skeletal muscle shapes postprandial metabolism, drives diverse cardiometabolic phenotypes and reveals potential therapeutic targets for nutritional intervention. The gut microbiome is another biological variable closely related to postprandial metabolism. In a recent study of 1000 healthy individuals, gut microbiome composition explained 7.5% of the interindividual variability in circulating triglyceride concentrations following a high fat meal challenge [124]. Although modest, this was greater than the estimated contributions from sex (~6%), previous meal composition (<4%), or genetics (<1%). Large-scale studies also support an association between gut microbiome composition and the chronic progression of obesity-related disease [125]. Importantly, whereas microbial signatures correlate only modestly with obesity risk *per se* [126], indices of microbial composition or richness appear more closely related to specific markers of metabolic dysfunction [127, 128]. Despite an abundance of studies revealing correlations between the gut microbiota and features of human disease, evidence to date for a direct causative role of the gut microbiome in obesity-related disease remains scant. Nevertheless, microbiota transfer (via fecal transplant) from humans with obesity recapitulates many aspects of donor-specific (patho)physiology in recipient mice [129, 130], hinting at a causal link between dysbiosis of the gut microbiome and obesity-related disease progression.

The observation that heterogeneity in the gut microbiota is largely confined to discrete clusters, or enterotypes [131], provides a means of stratifying patients with obesity by microbiome phenotypes. For example, the recent Metacardis study demonstrated that participants of the enterotype *Bacteroides* 2 (Bact2), characterised by a low-microbial diversity, displayed pronounced systemic inflammation [132] and an increased risk of cardiometabolic disease progression [133] compared to non-Bact2 participants. Importantly, habitual diet appears to play a significant role in determining enterotypes in adults, with Bact enterotypes being closely associated with a higher intake of protein and animal fats [134, 135]. Conversely, non-Bact enterotypes (i.e. *Prevotella*) have been linked to a greater carbohydrate and fibre intake from fruit and vegetables [135].

### Enterotypes predict response to dietary intervention

There is also growing appreciation for the role of enterotypes in predicting responses to dietary intervention. For example, caloric restriction has been shown to enhance microbial diversity in individuals with obesity, with these effects being especially prominent in Bact2 enterotypes [135, 136]. Conversely, non-Bact individuals with obesity (here categorised as having a higher *Prevotella* / *Bacteroides* ratio) were found to lose more weight than individuals with a lower *Prevotella* / *Bacteroides* ratio during a 24-week fibre-rich diet intervention [55, 137]. Similarly, in a small cohort of 49 individuals with obesity or overweight, improvements in systemic and adipose inflammation following short-term caloric restriction were only observed in individuals with a higher gene richness at baseline [135]. Thus, evidence to date suggests that high fibre diets could be particularly beneficial in *Prevotella* enterotypes, whereas Bact enterotypes appear more refractory to the metabolic benefits of increased fibre intake and may instead benefit from targeted approaches to increase microbiota diversity [138].

Although the mechanisms through which different enterotypes influence dietary responses and metabolic health are only beginning to be understood, emphasis has been placed on the microbial metabolites produced by different commensal communities. Notably, short chain fatty acids (SCFA) have been highlighted as potential transducers of the health benefits of increasing fibre intake in *Prevotella* enterotypes [139, 140], prompting speculation that SCFA supplementation could offer a strategy to overcome the poor fibre-metabolising characteristics of Bact2 enterotypes. A recent study demonstrated the feasibility of a novel nutritional approach to increase systemic SCFA availability whilst circumventing the poor tolerability of SCFA supplements [141], which may prove a useful tool for the future development of enterotype-guided precision nutrition. Finally, oral supplementation of certain pasteurized commensal bacteria may offer another means of harnessing the metabolic benefits of healthy enterotypes. For example, daily ingestion of *Akkermansia muciniphila* for three months was recently shown to improve indices of metabolic health (insulin sensitivity, plasma lipid profile and fat mass) in individuals living with overweight or obesity during a randomised, double-blind, placebo-controlled trial [142].

## Innate immune dysfunction to guide precision nutrition

Chronic low-grade inflammation, typified by innate immune dysfunction and increased systemic concentrations of pro-inflammatory markers, is another hallmark of obesity that interacts with insulin resistance and microbial dysbiosis to further exacerbate metabolic disease [143]. Cell surface marker analyses of peripheral blood immune cells indicates that metabolic dysfunction in individuals with obesityis associated with a shift towards a chronically activated, proinflammatory phenotype of circulating monocyte pools [144, 145]. Whether systemic low-grade inflammation defines another pathophysiological subset of individuals with obesity, or is an inevitable consequence of chronic weight gain, remains unclear.

Postprandial changes in circulating inflammatory markers (e.g. cytokines) are often used to assess interactions between diet and innate immune function. Most recently the change in acetylated glycoproteins, a purported composite biomarker of low-grade inflammation [146], was found to correlate positively with postprandial triglycerides, visceral adiposity and indices of poor microbial diversity following a mixed-meal challenge in >1000 individuals [147]. Similarly, we have previously illustrated that the inflammatory response to a high-fat challenge is closely related to numerous cardiometabolic risk modifiers, including BMI, age, HOMA-IR and gender [148–150]. However, the numerous immunomodulatory triggers to which circulating cells are exposed in obesity include nutrient-mediated signals from the adipose tissue (e.g. cytokine release), liver (e.g. acute phase protein response) and gut (e.g. microbial metabolites). As such, current measures of postprandial inflammation in obesity reflect a complex integrative picture of multi-tissue dysfunction and their interpretation with respect to identifying targetable pathophysiologic pathways remains extremely challenging.

Treating innate immune dysfunction as a modifiable phenotype in obesity is problematic due to its overlapping pathophysiology with other features of metabolic disease, together with complexities in defining and measuring chronic low grade inflammation [151]. Dietary intervention studies in humans rely largely on reductions in isolated plasma cytokine markers of inflammation status, most commonly CRP, TNFα, IL-6, IL-1β and IL-1RA. Reductions in one or more of these biomarkers have been reported following caloric restriction [152], time restricted eating [153, 154] and high fiber diets [155], yet results are very inconsistent across studies. Whilst these biomarkers may be closely associated with changes in body weight and inflammation [151], they provide little information about disease mechanisms.

The recent (re)emergence of immunological memory as a biologically-salient feature of the innate immune system [156] has led to speculation that adverse dietary triggers could pathologically “train” circulating cells, such as monocytes and neutrophils, to exaggerate postprandial inflammation and thus accelerate the progression of obesity-related disease [157]. Such a paradigm offers exciting potential for the targeted resolution of chronic inflammation in the precision nutrition space and warrants further translational investigation to advance this concept beyond existing preclinical model systems. It is important to appreciate that the epigenetic reprogramming of innate immune cells following pathogenic training is only functionally observable following subsequent restimulation, for example with lipopolysaccharide [158]. Capturing the phenotypic consequences of innate immune training in humans thus requires us to move past the measurement of systemic cytokine concentrations, perhaps instead adopting standardized and scalable assays of *ex vivo* innate immune stimulation [159].

## Precision nutrition to compliment adjunct pharmacotherapies

In terms of successful treatment options, we need to integrate precision nutrition with precision medicine to a greater extent and, indeed, there are a few potential candidates that require further investigation. With regards to targeting mechanisms beyond weight loss, much can be learned from the insulin sensitizing drug pioglitazone. Predominantly a PPARγ ligand, pioglitazone reduces visceral fat and ectopic lipid content despite increasing subcutaneous adiposity [160], which underscores the importance of PPARγ-mediated adipose expansion in metabolic health. Such pharmacotherapies also provide a useful research tool for the identification of future precision nutrition targets. For example, we recently observed a consistent remodeling of cell membrane lipids in adipose tissue from individuals with obesity treated with pioglitazone [161]. Specifically, pioglitazone treatment lowered the abundance of plasmalogen glycerophospholipids, which act as important regulators of cell membrane fluidity and have previously been implicated in restricting adipose tissue expansion in extreme obesity [162]. Moreover, pioglitazone selectively lowered the enrichment of the proinflammatory omega-6 lipid arachidonic acid in adipose cell membranes, without altering levels of omega-3 docosahexaenoic acid, providing a novel mechanistic link between adipose expandability and systemic inflammation. Whether prospective natural ligands of PPARγ, such as branched chain fatty acids [163], represent a useful nutritional approach to combat visceral adiposity, especially in MUO phenotypes, requires further study.

In addition to insulin-sensitizing therapeutics like pioglitazone, the recent repurposing of other type 2 diabetes medications for weight management heralds a new optimism for obesity treatment. Pivotally, the glucagon-like peptide one (GLP-1) receptor agonists received approval for chronic weight management following phase three clinical trials demonstrating average weight losses of ~15% [164, 165]. Furthermore, recent weight loss data following treatment with dual [7] and triple [166] incretin mimetic drugs appears to be even more impressive. That this extent of weight loss might be achieved safely with pharmacotherapeutics raises important questions for the field of precision nutrition. Firstly, can we develop nutritional strategies targeting similar mechanisms of action, albeit with lower efficacy? This is crucial since, despite their effectiveness, the likely cost of newer generations of weight loss medications may restrict their use to certain countries or patient populations [167].

Secondly, there is a vast opportunity to define if precision nutrition approaches can be used as an adjunct therapy alongside incretin mimetic medications, which allow patients to control appetite, and therein perhaps to potentiate responses in certain individuals. Indeed, the efficacy of incretin mimetic therapies remains highly variable. For example, following two years of Semaglutide (a GLP-1 receptor agonist) treatment, 36% of individuals lost >20% body weight, but an approximately equal fraction (38%) lost <10% weight [165]. Moreover, Semaglutide-mediated reductions in body weight do not appear effective in reversing all obesity-related maladies, such as liver fibrosis [104], reinforcing the need for complementary therapies that target mechanisms independent of weight loss. Understanding the biological factors underlining heterogeneity in these treatment responses can further inform on the most appropriate dietary strategies needed to compliment pharmacotherapy.

## Conclusions

Large scale impacts of precision nutrition require the integration of obesity surveillance programmes, that identify sub-cohorts (or distinct metabolic phenotypes) within populations at the greatest risk of disease progression, with tailored treatment paradigms that incorporate patient heterogeneity to optimise health benefits. Significant progress has been made in understanding how phenotypic differences in the pathophysiology of obesity contribute to heterogenous disease progression, but we are only now beginning to probe how obesity phenotypes influence responses to dietary intervention and might therefore be used to guide personalised nutrition. Current focus on understanding the ‘diet-sensitive metabolic nodes’ which render individuals more sensitive to the removal of adverse dietary stimuli (e.g. saturated fats, fructose, cholesterol), or most likely to benefit from nutritional modulation of the innate immune response (e.g. through pro-resolving or anti-inflammatory diets), represents a promising avenue for future progress. However, numerous conceptual and practical hurdles persist in translating metabolic phenotypes into useful clinical guidelines for precision nutrition approaches. Although beyond the scope of this review, the importance of behavioural, psychological and sociocultural factors in dietary prescription should not be underestimated, since these encompass the central determinants of patient adherence [168]. Transdisciplinary studies that integrate both patient physiology and psychosocial phenotypes into predictive models are likely to further improve the efficacy of tailored nutritional strategies to combat obesity-related disease.
